# Long inverted repeats around the chromosome replication terminus in the model strain *Bacillus thuringiensis* serovar *israelensis* BGSC 4Q7

**DOI:** 10.1099/mgen.0.000468

**Published:** 2020-11-12

**Authors:** Alexander Bolotin, Benoit Quinquis, Hugo Roume, Michel Gohar, Didier Lereclus, Alexei Sorokin

**Affiliations:** ^1^​ Micalis Institute, INRAE, AgroParisTech, Université Paris-Saclay, 78350 Jouy-en-Josas, France; ^2^​ MGP, INRAE, Université Paris-Saclay, 78350 Jouy-en-Josas, France

**Keywords:** *Bacillus thuringiensis*, chromosome structure, replication termination, long inverted repeats, plasmid-chromosome association, BGSC strains

## Abstract

*
Bacillus thuringiensis* serovar *israelensis* is the most widely used natural biopesticide against mosquito larvae worldwide. Its lineage has been actively studied and a plasmid-free strain, *B *. *thuringiensis* serovar *israelensis* BGSC 4Q7 (4Q7), has been produced. Previous sequencing of the genome of this strain has revealed the persistent presence of a 235 kb extrachromosomal element, pBtic235, which has been shown to be an inducible prophage, although three putative chromosomal prophages have been lost. Moreover, a 492 kb region, potentially including the standard replication terminus, has also been deleted in the 4Q7 strain, indicating an absence of essential genes in this area. We reanalysed the genome coverage distribution of reads for the previously sequenced variant strain, and sequenced two independently maintained samples of the 4Q7 strain. A 553 kb area, close to the 492 kb deletion, was found to be duplicated. This duplication presumably restored the equal sizes of the replichores, and a balanced functioning of replication termination. An analysis of genome assembly graphs revealed a transient association of the host chromosome with the pBtic235 element. This association may play a functional role in the replication of the bacterial chromosome, and the termination of this process in particular. The genome-restructuring events detected may modify the genetic status of cytotoxic or haemolytic toxins, potentially influencing strain virulence. Twelve of the single-nucleotide variants identified in 4Q7 were probably due to the procedure used for strain construction or were present in the precursor of this strain. No sequence variants were found in pBtic235, but the distribution of the corresponding 4Q7 reads indicates a significant difference from counterparts in natural *B. thuringiensis* serovar *israelensis* strains, suggesting a duplication or over-replication in 4Q7. Thus, the 4Q7 strain is not a pure plasmid-less offshoot, but a highly genetically modified derivative of its natural ancestor. In addition to potentially influencing virulence, genome-restructuring events can modify the replication termination machinery. These findings have potential implications for the conclusions of virulence studies on 4Q7 as a model, but they also raise interesting fundamental questions about the functioning of the *Bacillus* genome.

## Data Summary

Illumina sequencing reads, corresponding to the *Bacillus thuringiensis* serovar *israelensis* strain samples AM65-52, ATCC 35646, 4Q7_AS_ and 4Q7_JM_, were deposited in National Center for Biotechnology Information (NCBI) database (https://www.ncbi.nlm.nih.gov/): as BioProject PRJNA303961 (https://www.ncbi.nlm.nih.gov/bioproject/?term=PRJNA303961) under SRR8467560 (https://trace.ncbi.nlm.nih.gov/Traces/sra/?run=SRR8467560), SRR8474067 (https://trace.ncbi.nlm.nih.gov/Traces/sra/?run=SRR8474067), SRR11567778 (https://trace.ncbi.nlm.nih.gov/Traces/sra/?run=SRR11567778) and SRR11565157 (https://trace.ncbi.nlm.nih.gov/Traces/sra/?run=SRR11565157), respectively. Oxford Nanopore Technologies (ONT) sequencing reads for *B. thuringiensis* serovar *israelensis* 4Q7_JM_ were deposited as SRR11575654 (https://trace.ncbi.nlm.nih.gov/Traces/sra/?run=SRR11575654). A version of the 4Q7_JM_ genome sequence consisting of two contigs, automatically assembled from Illumina and ONT reads with Unicycler software, was deposited under accession numbers CP051858 (https://www.ncbi.nlm.nih.gov/nuccore/CP051858) and CP051859 (https://www.ncbi.nlm.nih.gov/nuccore/CP051859).

The sequencing reads SRR1174235 (https://trace.ncbi.nlm.nih.gov/Traces/sra/?run=SRR1174235) and assembly deposited under accession no. GCA_000585975.1 (https://www.ncbi.nlm.nih.gov/assembly/GCA_000585975.1) in BioProject PRJNA238495 (https://www.ncbi.nlm.nih.gov/bioproject/PRJNA238495), corresponding to sequencing data for the strain sample referred to here as *B. thuringiensis* serovar *israelensis* 4Q7_KBC_, and reported in [1], and the set of reads DRR002381 (https://trace.ncbi.nlm.nih.gov/Traces/sra/?run=DRR002381) in BioSample SAMD00015926 of BioProject PRJDB2767 (https://www.ncbi.nlm.nih.gov/bioproject/247315), corresponding to the strain *B. thuringiensis* LDC-391 cytotoxic to human cancer cells [2, 3], were downloaded from the NCBI server (https://www.ncbi.nlm.nih.gov/).

**Impact Statement**

We report a reanalysis of the *Bacillus thuringiensis* serovar *israelensis* BGSC 4Q7 genome based on previous and newly generated data for three independently maintained samples of the strain. Genome assembly from both short and long reads revealed the presence of extraordinarily long inverted repeats in the vicinity of the chromosome replication terminus. One plasmid persisted after the strain had been cured of all other plasmids. Our data indicate that this extrachromosomal element, pBtic235, formed a transient physical association with the bacterial chromosome, but not through simple prophage-like or transposon insertion. Instead, the inverted repeats were extended, probably rendering the chromosome linear. These data suggest the possible involvement of the pBtic235 element in bacterial chromosome maintenance via participation in the termination of chromosomal replication, compensating for the loss of chromosomal function and accounting for the persistence of pBtic235 in strains cured of other plasmids. This model genome sheds new light on the possible functions of large plasmids in bacteria. In particular, if strains are cured of the plasmids for use as model organisms, the integrity of their genomes relative to their plasmid-containing predecessors should be carefully checked. Further studies are required, with modern sequencing technologies, to elucidate the exact structure of the genome of the *B. thuringiensis* serovar *israelensis* 4Q7 strain.

## Introduction

Several lineages of the species *Bacillus thuringiensis* are widely used as non-hazardous biopesticides [4]. The lineage designated *B. thuringiensis* serovar *israelensis* is of particular importance, because some of its members are highly effective against mosquito larvae [5]. This lineage has been actively studied since its discovery in 1976 [6], with characterization of the molecular agents active against mosquito larvae [7, 8] and the transfer of plasmids [9–11] in particular. A series of strains has been constructed in which all plasmids have progressively been eliminated, including those responsible for insecticidal activity and conjugation [10]. In particular, the *B. thuringiensis* serovar *israelensis* 4Q7 strain was thought to have been cured of all the plasmids originally present in this lineage. However, this strain was recently characterized further by draft genomic sequencing [1], which revealed the presence of a remnant plasmid, pBtic235, which was subsequently shown to be an inducible prophage [12, 13]. The important chromosomal modification identified in this strain was a deletion of about 492 kb in the area including the replication terminus [1]. However, a comparison of the assembled contigs for this strain with those of other *B. thuringiensis* serovar *israelensis* strains revealed a significant difference in total size that could not be readily explained without detailed scrutiny of the original raw sequencing data [12]. Three predicted chromosomal prophages were found to be absent in *B. thuringiensis* serovar *israelensis* 4Q7, accounting for 120 kb [14]. We investigated the differences between the *B. thuringiensis* serovar *israelensis* 4Q7 and the genomes of other members of the lineage in more detail, with a view to elucidating the reasons for differences in genome size, by reanalysing the raw sequencing data deposited in the National Center for Biotechnology Information (NCBI) database by Jeong *et al*. [1], and analysing sequencing data generated here for two other samples of this strain that had been maintained independently for more than 20 years. The analysis was based on a small number of available complete genome sequences for this lineage [12, 15, 16]. We identified additional features in the *B. thuringiensis* serovar *israelensis* 4Q7 genome, raising questions not only about fundamental aspects of chromosome maintenance, but also about the suitability of this strain as a model for this important bacterium.

## Methods

### Samples of the *B. thuringiensis* serovar *israelensis* 4Q7 strain and sources of raw sequencing data

Two laboratory samples of the *B. thuringiensis* serovar *israelensis* 4Q7 strain were used for DNA preparation for this study. These samples were obtained from the *Bacillus* Genetic Stock Center (BGSC, Columbus, USA) by A. Sorokin and J. Mahillon (Louvain-la-Neuve, Belgium). They had been maintained independently for at least 25 years, and are labelled here as *B. thuringiensis* serovar *israelensis* 4Q7_AS_ and 4Q7_JM_, respectively. DNA was prepared from these strains as previously described [12], and sent to Eurofins GATC Biotech for the production of standard genomic DNA-fragment libraries and sequencing reads on a HiSeq platform (Illumina). For *B. thuringiensis* serovar *israelensis* 4Q7_JM_, the same DNA preparation was used to produce a DNA-fragment library for a MinION run and base-calling for the generation of a fastq file, in accordance with the manufacturer’s protocol (Oxford Nanopore Technologies; ONT). The SRR1174235 sequencing reads, corresponding to a sequencing experiment reported in [1] for *B. thuringiensis* serovar *israelensis* 4Q7_KBC_, were downloaded from the NCBI server. A set of SRR data for 91 *B. thuringiensis* and 480 *Bacillus cereus* genomes was also retrieved from the NCBI database, with the species names used as keywords. The genomic sequence of the *B. thuringiensis* serovar *israelensis* AM65-52 strain used for template-assisted assembly (variation analysis) was reported in a previous study [12]. The same DNA preparations for the *B. thuringiensis* serovar *israelensis* AM65-52 and ATCC 35646 strains, stored at −20 °C for 2 years, were sent to Eurofins GATC Biotech for the production of standard genomic DNA-fragment libraries and sequencing reads on a HiSeq platform (Illumina). The read data for the *B. thuringiensis* serovar *israelensis* ATCC 35646 strain were generated in two sequencing campaigns, in 2017 and 2019. We found several differences in the distribution of their alignments over the template sequence, as illustrated in Fig. S1 (available with the online version of this article). It has been reported that the apparent shape reproducibility of random read distributions may be related to the local G+C-content of the genomes [17, 18]. This issue, related to the DNA-fragment library preparation or base-calling protocol, does not influence the conclusions of our work. The newly generated reads were used to generate control read alignments. The main read alignment results obtained for the *B. thuringiensis* serovar *israelensis* 4Q7_KBC_ sample (accession no. SRR1174235) were confirmed with the Illumina data for the 4Q7_AS_ and 4Q7_JM_ samples. The minor differences between the three samples of strain 4Q7 are discussed in Results.

### Data analysis and release

**Fig. 1.**
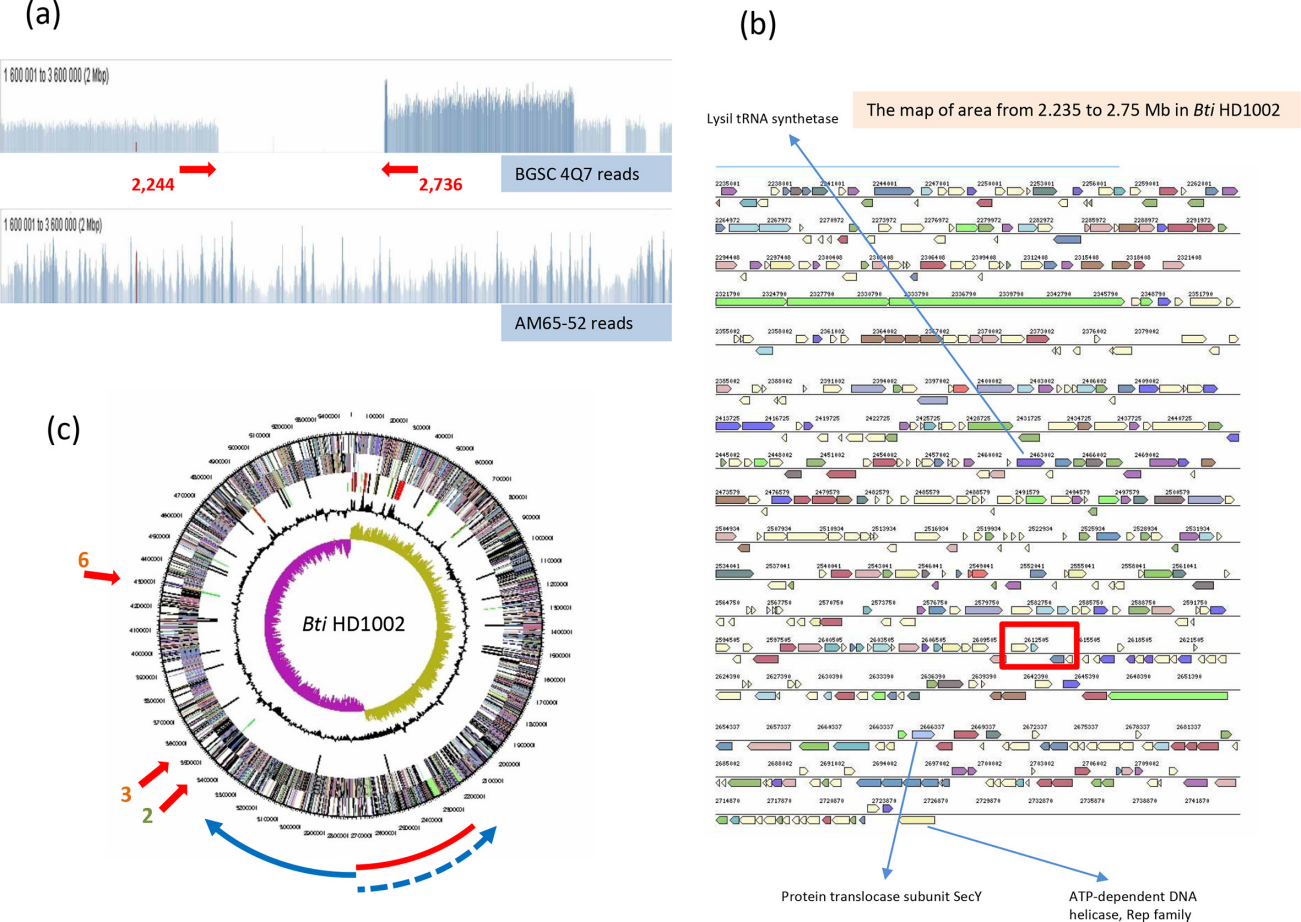
Deletions and duplication on the *B. thuringiensis* serovar *israelensis* 4Q7 chromosome. (a) Distribution of Illumina sequencing reads, in coverage per nucleotide, for the 4Q7 (top) and AM65-52 (bottom) strains, over the AM65-52 genome (accession no. CP013275). The 492 kb deletion in 4Q7, corresponding to the 2244 to 2736 kb positions in AM65-52, is indicated by red arrows. Read coverage is higher for the duplicated 553 kb area from 2745 to 3298 kb. The distribution image was copied from the Tablet interface panel and modified slightly to improve its readability. The vertical and horizontal scales are linear, and the values are not shown. The distribution shown corresponds to assembly from the SRR1174235 sequencing reads [1]. A very similar distribution was also obtained with the reads for the 4Q7_AS_ and 4Q7_JM_ samples generated in this study (accession numbers SRR11567778 and SRR11565157; not shown). (b) Coding sequence (CDS) map of the 2.235 to 2.75 Mb region of the *B. thuringiensis* serovar *israelensis* HD1002 strain corresponding to the 492 kb deletion in 4Q7. Potentially essential genes are indicated by blue arrows. The red rectangle indicates the region close to 2622 kb in which GC-skew and the preferential orientations of CDS change sign, which is deleted in 4Q7. The 492 kb deletion includes the *dif* site (5′-CCTATAATATATATTATGTTAACT-3′) mapping to this area [32]. (c) Circular map of the *B. thuringiensis* serovar *israelensis* HD1002 chromosome. The circles from the centre represent: 1, GC-skew; 2, G+C content distribution; 3, positions of repeated elements; 4 and 5, CDS in the anticlockwise and clockwise directions, respectively; 6, position scale for the circular genome. Red arrows indicate the locations of prophages deleted in the 4Q7 strain. The red segment indicates the 492 kb deletion, and the blue solid arrow indicates the area duplicated in this strain, the potential second copy is indicated by a dashed arrow. The source of information for the HD1002 strain and figure design for (b) and (c) are from the IMG database of the DOE Joint Genome Institute [28]. *Bti*, *B. thuringiensis* serovar *israelensis*.

SPAdes [19], bwa-mem [20] and Unicycler [21] tools implemented on the patric [22] and Galaxy [23] servers or locally installed versions (SPAdes, 3.13.0; Unicycler, 0.4.7; canu, 1.6; Shasta, 0.1.0; bwa, 0.7.12-r1039; bowtie2, 2.2.6; minimap2, 2.17-r941; SAMtools, 1.9; vcftools, 0.1.13) were used for the *de novo* assembly and/or template-assisted mapping of sequencing reads. The local versions were mostly used through the INRAE *MIGALE* bioinformatics platform (http://migale.jouy.inra.fr). File contents were visualized and sequence assemblies were analysed with Tablet (version 1.16.09.06) [24], CGView (v. 1.7) [25] and Bandage (v. 0.8.1) [26]. Statistical data were processed and visualized with R software (v. 3.6.1) [27]. Screen shots of the read distribution produced by these programs have been provided to illustrate our findings. The genomic sequence of the *B. thuringiensis* serovar *israelensis* HD1002 strain (accession no. NZ_CP009351), which is very closely related to AM65-52 (accession no. CP013275), was used to determine the positions of deletions and duplications (Fig. 1b, c), this sequence being present in the IMG (Integrated Microbial Genomes) database [28]. The HD1002 sequence presented in IMG was re-annotated, and its orientation and starting position were modified relative to the NZ_CP009351 sequence, according to the GenBank entry for AM65-52. The newly produced sequencing reads, corresponding to the AM65-52 and ATCC 35646 strains, and the 4Q7_AS_ and 4Q7_JM_ samples of the 4Q7 strain used here, were deposited as BioProject PRJNA303961 under accession numbers SRR8467560, SRR8474067, SRR11567778 and SRR11565157, respectively. ONT sequencing reads for the 4Q7_JM_ sample were deposited under accession no. SRR11575654. The corresponding statistical data can be retrieved under these accession numbers. A version of the 4Q7_JM_ genome sequence, consisting of two contigs automatically assembled from Illumina data and part of the ONT read set, obtained with Unicycler software, was deposited under accession numbers CP051858 and CP051859.

## Results

### Conflicts between the sizes of the *de novo* assembly of 4Q7 Illumina reads and complete genome sequences

A comparison of the total length of the contigs available from the NCBI database for the *B. thuringiensis* serovar *israelensis* 4Q7 strain (5.04 Mb, assembly accession no. GCA_000585975.1) with the completely assembled sequences of the chromosomes of the same subspecies (5490 to 5500 kb) [12] revealed a large difference in size, of about 500 kb, that requires explanation, although the error on this estimate may be as large as 20 kb, due to problems assembling rRNA-encoding regions or other complex repeats. We first confirmed this finding by re-assembling the sequence five times *de novo*, varying the assembly parameters in SPAdes. We obtained very similar results (5040±0.010 kb for 45 to 65 contigs with a length greater than 300 bp). Three main features of this sequence that could potentially account for the difference in genome size between 4Q7 and its relatives had already been detected. The first of these features is a 492 kb deletion corresponding to the positions between 1783 and 2275 kb of the *B. thuringiensis* serovar *israelensis* HD-789 genome (accession no. CP003763; or 2244 to 2736 in AM65-52, accession no. CP013275; Fig. 1a), used as a reference [1]. The second feature identified, the pBtic235 plasmid, which remains present in *B. thuringiensis* serovar *israelensis* 4Q7, provides an additional 235 kb, thereby decreasing the total estimated size of the precursor chromosomal contigs to 5297 kb (5040+492–235). The third feature identified is the three chromosomal prophages recently detected and named regions 2, 3 and 6, at 3407 to 3452 kb, 3511 to 3552 kb and 42 778 to 4319 kb, respectively, in the *B. thuringiensis* serovar *israelensis* AM65-52 genome (Fig. 1). These prophages account for a total of 127 kb (45+41+41). All three prophages are absent from the 4Q7 chromosome [14]. Taking these deletions and the identity of the 235 kb contig into account, about 76 kb of the consensus 5500 kb chromosome of the lineage remain absent and unaccounted for in the 4Q7 strain assembly (5297+127=5424 kb). Assuming collinearity between the 4Q7 genome and the genomes of other *B. thuringiensis* serovar *israelensis* strains, another key issue that remains unresolved is how this bacterium stably maintains the apparent asymmetry of its chromosome following this large deletion. There is a very strong correlation between the sites at which GC-skew (G-C/G+C contents in a window) change sign and the positions of replication origins and termination sites in circular bacterial chromosomes. Thus, in terms of GC-skew or the majority orientation of protein-encoding genes, the chromosomes are usually symmetric around an axis linking the origin and termination sites of replication [29]. Given its strength, this correlation is probably of biological significance. Deleting 492 kb, mostly from one replichore, should lead to an imbalance in replication complex progression, potentially leading to other restructuring events, due to conflicts between replication and transcription complexes moving in opposite directions in particular [30, 31]. The deleted 492 kb region includes a site at which the sign of GC-skew changes and the *dif* site [32], corresponding to the region in which chromosomal replication is thought to terminate (Fig. 1b, c). The two replichores in the *B. thuringiensis* serovar *israelensis* genome are not of equal size (see Discussion), but a pure deletion of this magnitude would be expected to render cells containing the modified chromosome less viable.

### Template-assisted re-assembly of 4Q7 reveals considerable overcoverage of an area near the replication termination site

**Fig. 2.**
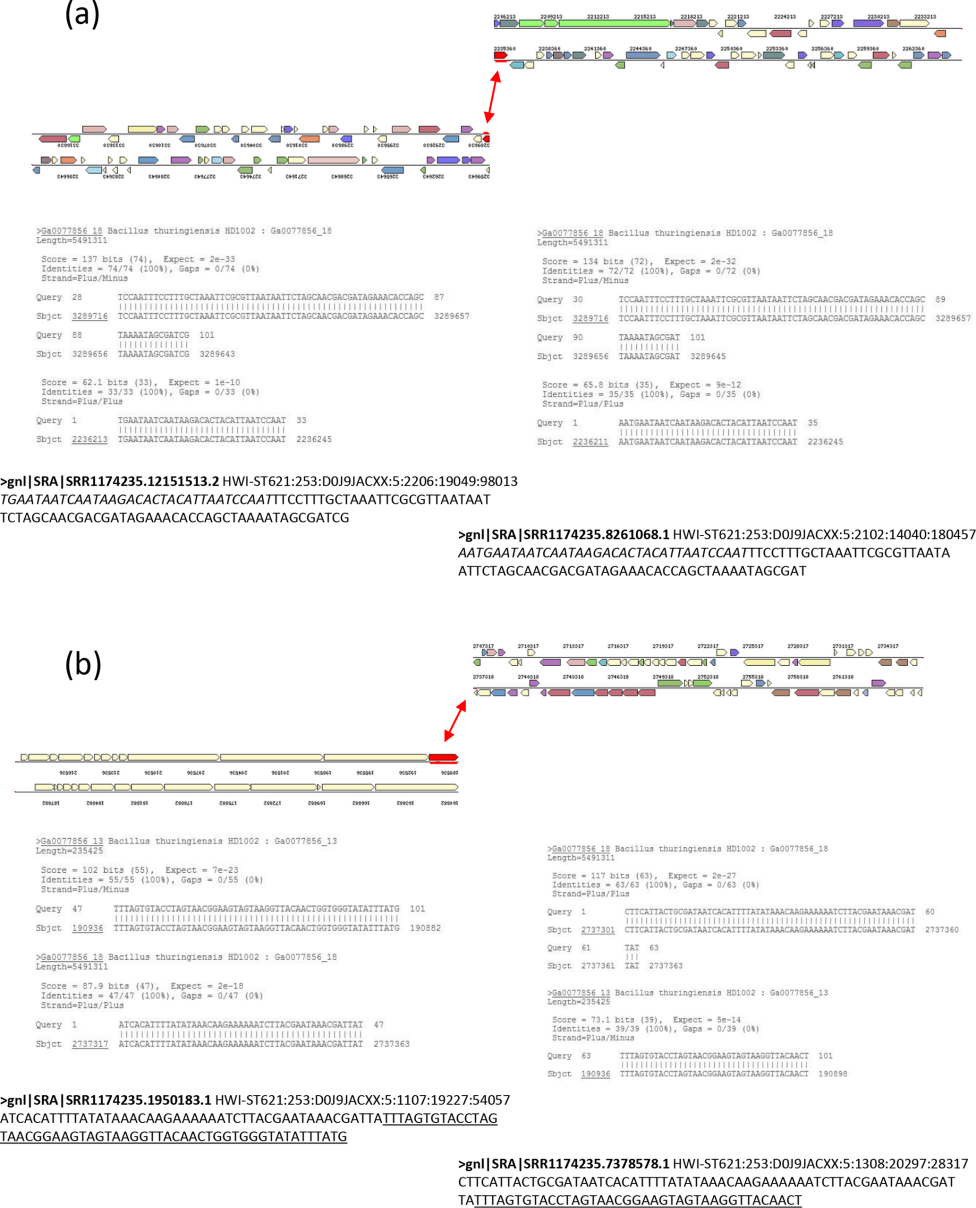
Examples of sequencing reads from SRR1174235 data confirming the inverted repeat and pBtic235-to-chromosome joins in the *B. thuringiensis* serovar *israelensis* 4Q7 strain. (a) Reads confirming the inverted repeat join. Sequences of two reads extracted from the SRR1174235 dataset for the 4Q7 strain are shown at the bottom. The corresponding BLASTn analysis against the *B. thuringiensis* serovar *israelensis* HD1002 genome is indicated in the middle. Each splitting of a read (corresponding to the 2 236 245 and 3 289 716 bp positions in HD1002) indicates the covalent join between two non-neighbouring template genome areas, shown at the top. Homology spots and their links are indicated by red bars and arrows. The two reads shown were randomly selected from about 420 confirming this link. The two sequences in the reads, non-adjacent in HD1002, are shown in italics and plain text. About 700 reads confirming the usual assembly link, corresponding to 3 289 716 bp in HD1002, for multiple *B. cereus* group genomes are also present in SRR1174235, but examples are not shown. (b) Reads confirming the joins between the chromosome and the pBtic235 element. As in (a), two reads extracted from SRR1174235 are shown at the bottom. The corresponding BLASTn analysis against the *B. thuringiensis* serovar *israelensis* HD1002 genome is indicated in the middle. Each splitting of a read indicates the covalent join between two non-neighbouring template genome areas, shown at the top, with the chromosomal part on the right and the pBtic235 part on the left. Homology spots and their links are indicated by red bars and arrows. The two reads shown were randomly selected from about 410 reads confirming the link. The sequences in the reads corresponding to pBtic235 are underlined, and those corresponding to the chromosome are not underlined. About 800 reads for pBtic235, and 900 reads for the chromosome, confirming the usual assembly for multiple *B. thuringiensis* serovar *israelensis* genome structures are also present in SRR1174235, but examples are not shown.

Template-assisted assembly, also referred to as mapping or alignment, of the *B. thuringiensis* serovar *israelensis* 4Q7 reads revealed a key clue to the reaction of the bacterium to the apparent disequilibrium of its genome. The 4Q7_KBC_ read data (accession no. SRR1174235) presented in Fig. 1(a) show that, close to the large 492 kb deletion, the coverage of the genome by sequencing reads approximately doubles. A similar increase in coverage was also observed with the Illumina reads produced for the 4Q7_AS_ and 4Q7_JM_ samples (Fig. S2). We interpret this overcoverage as indicating the presence of two copies of this 560 kb area (corresponding to the region from 2737 to 3298 kb in the closely related *B. thuringiensis* serovar *israelensis* AM65-52 strain) in the genome of 4Q7. Such a duplication would essentially result in an equalization of the sizes of the two replichores, because the new putative replication termination site must be close to the end of the duplicated area. The doubling of read coverage actually corresponded only to the 8 kb region between 2737 and 2745 kb (Fig. 1a), with a rapid increase from 1.2- to 2.0-fold coverage in the preceding 553 kb. We investigated the structure of this duplicated area, by scrutinizing assembly variants with Bandage software [26], which analyses different possibilities through visualization of the assembly graph (see the results below and Discussion). Examples of multiple reads (420 in total) corresponding to one third of the total reads mapping to this chromosomal area (about 1100), confirming the physical connection of unexpected DNA links in the *B. thuringiensis* serovar *israelensis* 4Q7 genome, are presented in Fig. 2(a). Therefore, we would interpret the total read data as indicating that the entire 553 kb region, from 2745 to 3298 kb, was duplicated as an inverted repeat (Fig. 1c). The 8 kb region underwent several restructuring events that we were able to resolve with Bandage (see below), but which could not be definitively validated in this study. Surprisingly, we also found multiple reads confirming the physical connection between chromosomal and pBtic235 DNA (Fig. 2b). However, the rest of the reads, again about two thirds (about 800) of the total mapping to this area (1200 reads) corresponded to this element being entirely assembled into a circular contig. Moreover, we have no strong evidence for the integration of all or part of this element into the chromosome. This event would produce two entry sequences for the element and the chromosome, but we detected only one for each. Taking this finding and the gradient distribution of reads over this area (Fig. 1a) into account, an alternative reasonable interpretation of our data would be that, in this strain, chromosomal replication is preferentially terminated at several different points, resulting in some over-replication of segments between 8 and 550 kb in size. In some cases, the DNA of pBtic235 seems to resolve chromosomal concatemers during the termination of replication. It should be noted that this read distribution, indicating duplication or over-replication of this chromosomal area, was not observed with reads corresponding to *B. thuringiensis* serovar *israelensis* ATCC 35646 (not shown) or AM65-52 (Fig. 1a). Duplication of the 553 kb region as an inverted repeat restores the GC-skew pattern symmetry (not shown); thus, optimizing the functioning of the replication machinery.

### Difference in read distribution corresponding to prophage pBtic235 in environmental and 4Q7 strains

**Fig. 3.**
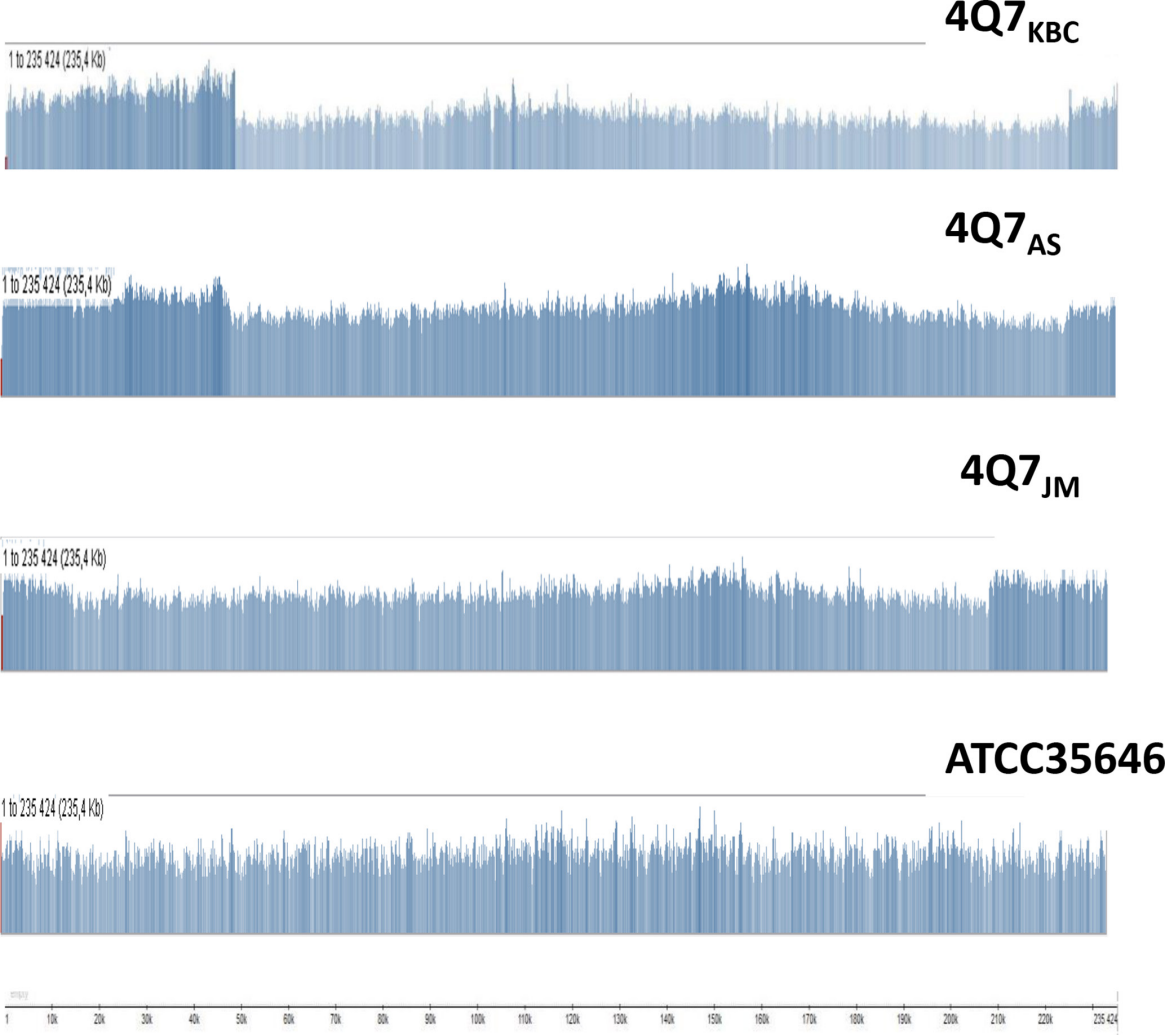
Coverage of the pBtic235 sequence with reads generated from DNA from different strains The distribution images were copied from the Tablet interface panel and have been modified slightly to improve readability. The reads for *B. thuringiensis* serovar *israelensis* 4Q7 strain samples (4Q7_KBC_, 4Q7_AS_ and 4Q7_JM_) and for the *B. thuringiensis* serovar *israelensis* ATCC 35646 strain, are shown from top to bottom. The vertical scales are linear, and the values are not shown. The horizontal scale, in kb, is drawn below the figure. The distribution for 4Q7_KBC_ corresponds to the sequencing reads from SRR1174235 [1]. Distributions for 4Q7_AS_, 4Q7_JM_ and ATCC 35646 were generated from the reads obtained in this study (accession numbers SRR11567778, SRR11565157 and SRR8474067, respectively).

The extrachromosomal genetic element pBtic235 appeared to be inducible and to be able to form biologically active phage [13]. It is the only extra-chromosomal DNA element remaining in *B. thuringiensis* serovar *israelensis* 4Q7 [12]. We compared the state of this element in the environmental and artificially modified strains described here, by aligning the Illumina reads for the *B. thuringiensis* serovar *israelensis* ATCC 35646 and 4Q7 strains, using the pBtic235 sequence of AM65-52 as a template (Fig. 3). The reads of AM65-52 were also used, but were omitted from Fig. 3 for the reasons explained in Methods (see also Fig. S1). Read coverage for 4Q7 samples was about 1.5 times higher in the areas extending from 1 to 48.6 and 225.5 to 235.4 kb for 4Q7_KBC_ and 4Q7_AS_, and in the areas extending from 1 to 15 and 210 to 235.4 kb for 4Q7_JM_ (Fig. 3). Moreover, in 4Q7_KBC_, in the position corresponding to 48 586 bp in pBtic235, multiple reads were split in two, upon alignment, with one part mapping to pBtic235 and the other to the chromosome, suggesting the existence of a covalent link between this element and the chromosomal DNA (Fig. 2b). A similar connection, described in more detail below, was detected for the *B. thuringiensis* serovar *israelensis* 4Q7_AS_ and 4Q7_JM_ samples. Interestingly, the copy number of pBtic235, estimated by read coverage (Table 1), was about 1.6 for all three samples of the 4Q7 strain, but only about 0.7 for all the environmental strains studied [12]. Nevertheless, it should be noted that the complete circular pBtic235 DNA was automatically assembled in one separate contig in our *de novo* assembly analysis, as in a previous study [12]. Moreover, this DNA was detected on electrophoresis and could be cured from the cells [13]. We currently have no reasonable explanation for this ambiguity. However, we cannot exclude the possibility that this element is integrated into the chromosome, rendering the chromosome linear, in some bacterial cells. As a precedent, an artificial stable linearization of the *Escherichia coli* chromosome mediated by incorporation of the N15 phage, was reported in a published study [33].

**Table 1.** pBtic235 element copy number in different samples of *B. thuringiensis* serovar *israelensis* 4Q7 and in environmental strains (AM65-52, ATCC 35646 and BMP144)

Strain sample	4Q7_KBC_	4Q7_AS_	4Q7_JM_	AM65-52	ATCC 35646	BMP144
Plasmid reads	1 903 671	698 236	416 587	202 988	830 518	1 070 076
Chromosomal reads	29 299 563	10 122 752	5 640 342	7 914 340	26 877 218	32 319 992
Plasmid copy number*	1.52	1.61	1.73	0.60	0.72	0.77

*Plasmid copy number was calculated as (plasmid reads/plasmid size)/(chromosomal reads/chromosomal size), where plasmid size is 235 424 bp and chromosomal size is 5 499 731 bp.The data for the environmental strains were obtained from our previously published study [12].

### Single-nucleotide mutations and small deletions in samples of the 4Q7 strain

Alignment of the *B. thuringiensis* serovar *israelensis* 4Q7 reads on the genomic sequence of strain AM65-52, used as a template, revealed 26 small variations (Table 2). We have excluded from Table 2 all regions containing non-perfect and other complex repeats, such as rRNA or tRNA gene clusters, for which ambiguity may be due to incorrect assembly rather than real variation between strains. Therefore, we considered only regions unique within the genome or perfectly repeated. We compared these 26 locations with other sequenced *B. thuringiensis* serovar *israelensis* strains, to reveal the variants specific to the studied samples of the 4Q7 strain. We found that 19 variants were present only in 4Q7 and, therefore, were acquired during its construction or maintenance, or were specific to the precursor, which is probably the *B. thuringiensis* serovar *israelensis* 4Q2 or HD500 strain [34, 35]. Twelve of these nineteen variants were present in all three samples of 4Q7 studied and, thus, either appeared during the construction of this strain or were present in its precursor. Seven variants were also present in several other *B. thuringiensis* serovar *israelensis* strains and, therefore, were simply specific to AM65-52, used as a template for the read alignment. Interestingly, seven other variants were present in only one of the 4Q7 samples: three in 4Q7_JM_ and four in 4Q7_AS_. These variants probably arose during maintenance of the strain samples, because the two samples were received from the BGSC more than 25 years ago. The 4Q7_KBC_ sample displayed no unique variation, presumably because it was obtained from the BGSC very recently, specifically for the genomic sequencing experiment [1]. The spontaneous mutation at 115 294 bp in 4Q7_JM_ confers the Rif^R^ phenotype selected for conjugation experiments. Three of the four variants in the 4Q7_AS_ sample were located in the highly mutable BclA-encoding gene, which contains many short repeats.

**Table 2.** Summary of differences between the *B. thuringiensis* serovar *israelensis* BGSC 4Q7 strain and AM65-52

Position in AM65-52	Nucleotide change	Amino acid change	Annotation by RAST	Variation in samples of 4Q7*	Variation in other *B. thuringiensis* serovar *israelensis* strains
50 293	75 bp del	25 aa del	DNA-binding protein SpoVG	AS – yes; KBC, JM – no	ATCC 35646, HD1002, HD789 – no
115 294†	A→G	Glu→Arg	DNA-directed RNA polymerase beta subunit	JM – yes; KBC, AS – no	ATCC 35646, HD1002, HD789 – no
564 011	T→del	Frameshift	Glycine betaine transporter OpuD	KBC, AS, JM – yes	ATCC 35646, HD1002, HD789 – no
636 259	AT→del	No change	Intergenic	KBC, AS, JM – yes	ATCC 35646, HD1002, HD789 – yes
875 553	A→G	Arg→His	Hypothetical protein	KBC, AS, JM – yes	ATCC 35646, HD1002, HD789 – no
1 222 626	C→G	Ala→Pro	BclA protein	AS – yes; KBC, JM – no	ATCC 35646, HD1002, HD789 – no
1 222 630	C→G	No change	BclA protein	AS – yes; KBC, JM – no	ATCC 35646, HD1002, HD789 – no
1 222 638	87 bp del	29 aa del	BclA protein	AS – yes; KBC, JM – no	ATCC 35646, HD1002, HD789 – no
1 355 830	C→T	Ser→Pro	Bacitracin transporter BCRB	KBC, AS, JM – yes	ATCC 35646, HD1002, HD789 – no
1 529 720	G→A	No change	Inner spore coat protein CotD	KBC, AS, JM – yes	ATCC 35646, HD1002, HD789 – no
1 529 729	G→A	No change	Inner spore coat protein CotD	KBC, AS, JM – yes	ATCC 35646, HD1002, HD789 – no
2 764 501	G→A	Val→Ile	DedA family membrane protein	KBC, AS, JM – yes	ATCC 35646, HD1002, HD789 – yes
2 860 235	G→A	No change	Putative kinase	KBC, AS, JM – yes	ATCC 35646, HD1002, HD789 – yes
2 958 066	T→C	Ile→Met	Phosphohydrolase (MutT family protein)	KBC, AS, JM – yes	ATCC 35646, HD1002, HD789 – yes
3 148 593	A→C	Ile→Arg	Penicillin acylase II	KBC, AS, JM – yes	ATCC 35646, HD1002, HD789 – yes
3 174 075	A→del	Frameshift	Capsule biosynthesis protein CapA	KBC, AS, JM – yes	ATCC 35646, HD1002, HD789 – no
3 175 718	T→C	Asn→Ser	MFS-type transporter YfkF	KBC, AS, JM – yes	ATCC 35646, HD1002, HD789 – yes
3 229 841	A→G	No change	Intergenic	KBC, AS, JM – yes	ATCC 35646, HD1002, HD789 – no
3 904 125	C→T	Met→Ile	P-type Ca^2+^-transport ATPase	KBC, AS, JM – yes	ATCC 35646, HD1002, HD789 – no
4 018 377	C→del	No change	Intergenic	KBC, AS, JM – yes	ATCC 35646, HD1002, HD789 – no
4 231 259	G→C	Ala→Pro	Transcriptional regulator, AcrR family	KBC, AS, JM – yes	ATCC 35646, HD1002, HD789 – no
4 247 985	G→T	No change	Intergenic	KBC, AS, JM – yes	ATCC 35646, HD1002, HD789 – yes
4 573 149	A→del	No change	Intergenic	JM – yes; KBC, AS – no	ATCC 35646, HD1002, HD789 – no
4 747 124	9 bp del	3 aa del	VrrB protein	KBC, AS, JM – yes	ATCC 35646, HD1002, HD789 – no
4 787 070	A→T	Ile→Leu	Sporulation kinase	KBC, AS, JM – yes	ATCC 35646, HD1002, HD789 – no
4 811 263	A→C	Asn→His	Glycerate kinase	JM – yes; KBC, AS – no	ATCC 35646, HD1002, HD789 – no

*Variations specific to a particular sample of the 4Q7 strain are underlined.

†Rif^R^ mutation in the *B. thuringiensis* serovar *israelensis* 4Q7_JM_ sample.

### Sequencing of the 4Q7_JM_ sample with nanopore technology

In an effort to improve the assembly of the *B. thuringiensis* serovar *israelensis* 4Q7 strain sequence, we generated long sequencing reads for the 4Q7_JM_ sample with MinION technology (ONT). We generated an estimated 350 thousand reads of 100 to 162 755 bases in size (for the read size distribution see Fig. S3). The alignment of multiple reads with the assembled contigs for the *B. thuringiensis* serovar *israelensis* genome sequences indicated a mean identity of 95%, although similar comparisons with other *B. cereus* group genomes revealed identity levels of only 85–87 % (not shown). An illustration of ambiguities in the ONT reads that we used, relative to the Illumina reads, is shown in Fig. S4. We first attempted to produce a complete and clean 4Q7_JM_ sequence with our set of available ONT reads and canu software [36], which was designed to clean up assembled sequences using only error-prone long reads. The software produced very small numbers of contigs (no more than one), but estimation of assembled sequence quality by comparison with available *B. thuringiensis* serovar *israelensis* genomes nevertheless indicated an error level of 0.5–1 % (not shown), presumably due to a non-random distribution of ambiguities in the reads. Alternatively, we used both sets of data (Illumina and ONT) available for the 4Q7_JM_ sample, and Unicycler software [21], which uses the SPAdes algorithm [19], for the initial assembly graph, and long reads for the resolution of assembly ambiguities. Use of the entire set of ONT data generally resulted in the program crashing or aborting, presumably due to multiple ‘co-cloning’ ambiguities in the long reads, which we estimated at about 2 %. However, using cleaned data or randomly selected (for example, by read-size interval) sets of about 50 000 ONT reads, we consistently obtained the reproducible assembly of a linear chromosomal contig of 4 865 236 bp and a circular 235 425 bp element corresponding to pBtic235. ‘Consistently’ and ‘reproducible’ imply here that the use of several random ONT read sets produced contigs of identical sizes. However, as we demonstrated above in the analysis of read distributions and search for relevant joins, the 553 kb terminal sequence of the chromosomal contig should be duplicated, but the software never automatically generated both of the joins required. It was also unable to circularize the chromosomal contig.

An alignment of short Illumina reads over the chromosomal part of the sequence and the pBtic235 element indicated that the sequences of the *B. thuringiensis* serovar *israelensis* 4Q7_JM_ sample were slightly different from those of 4Q7_KBC_ and 4Q7_AS_, close to the site of replication termination on the chromosome and in the part of pBtic235 with overcoverage (Figs S1 and S2). In the chromosomal replication termination area, the distribution for 4Q7_KBC_ and 4Q7_AS_ revealed overcoverage for one clear region of about 8 kb, which was absent from the 4Q7_JM_ sample. Moreover, the Unicycler assembly generated from Illumina data for one of these samples and ONT data for 4Q7_JM_ together yielded a linear chromosomal contig, slightly variable in size, but about 4859 bp long, and a linear 8015 bp contig, absent from assembly for the 4Q7_JM_. However, our sequencing data could not resolve this area unambiguously for any strain sample or join it to the rest of the chromosome assembly. Interestingly, this 8 kb contig contains a gene encoding a RecQ-family helicase of potential relevance for the maintenance of genome stability. This gene was absent from the 4Q7_JM_ sample. Given that the 553 kb inverted repeat is located close to the replication termination area, we submitted the 4 865 236 bp linear chromosomal and 235 425 bp circular pBtic235 element contigs to the NCBI database under the accession numbers CP051858 and CP051859, respectively, as one of the best automatic assemblies of the *B. thuringiensis* serovar *israelensis* 4Q7_JM_ genome obtained with the available sequencing data.

### Expert-assisted analysis of assembly graphs suggests that there are linear forms of the 4Q7 genome

**Fig. 4.**
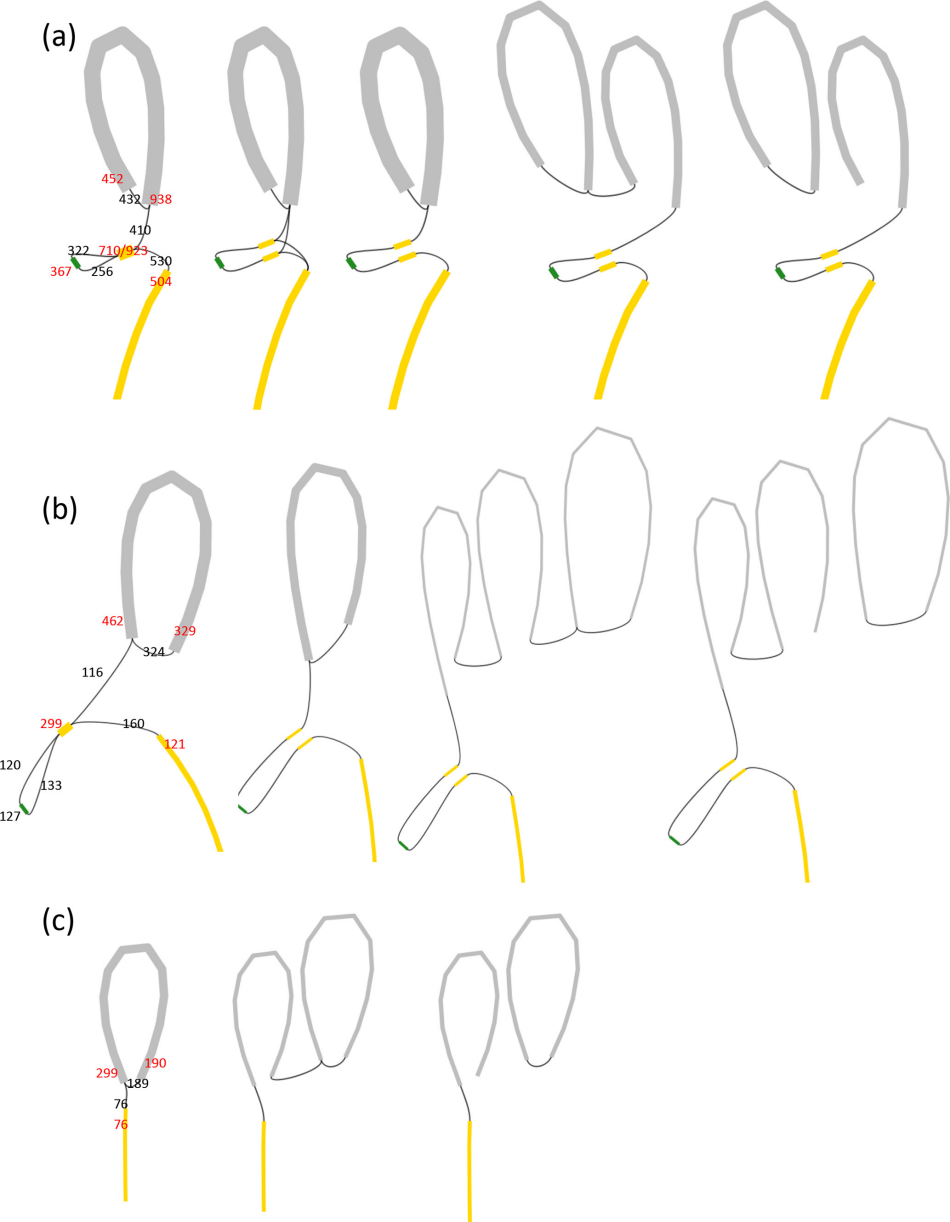
Bandage-assisted visualization of the assembly graphs and proposed resolution for the *B. thuringiensis* serovar *israelensis* 4Q7 genome. (a), (b) and (c) show the Bandage [26] visual presentation of the Bruijn graphs generated by the SPAdes assembler [19] for the 4Q7_KBC_, 4Q7_AS_ and 4Q7_JM_ samples, respectively. Curved grey, yellow and green lines, with thicknesses proportional to read coverage, represent nodes (contigs). Only the part of genome close to the replication termination area and the connection to the pBtic235 element node are shown. Thin black lines represent potential edges (links) that connect nodes, as proposed by the assembly software and corrected following scrutiny by an expert. The results of expert intervention are shown from the left to the right graphs. The red and black numbers on the cartoon on the left indicate the mean contig coverage and the number of reads supporting the edges proposed by the software, respectively. The graphs furthest to the right correspond to the best assembly based on expert scrutiny. Grey closed curved circular structures represent separate pBtic235 elements. The bacterial chromosome, thus, appears to be linear.

**Fig. 5.**
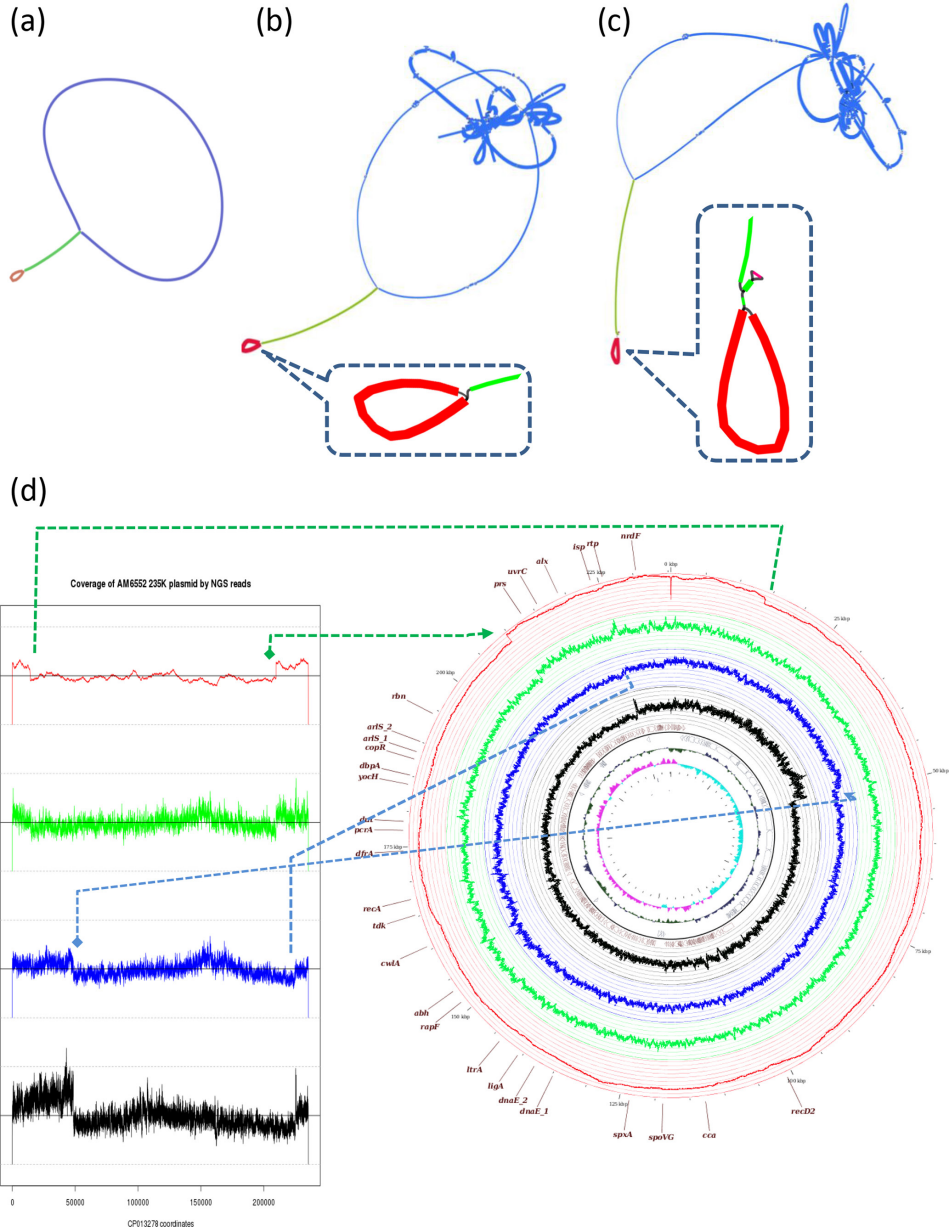
pBtic235 element-to-chromosome linkage on the genetic map of pBtic235. (a–c) Bandage presentation of assembly graphs for: *B. thuringiensis* serovar *israelensis* 4Q7_JM_ ONT reads assembled *de novo* with canu (a), 4Q7_JM_ Illumina reads assembled *de novo* with SPAdes (b) and 4Q7_AS_ Illumina reads assembled *de novo* with SPAdes (c). Blue indicates the chromosome nodes, apart from the long 553 kb repeats, which are shown in green. In (b) and (c), the complex repeats, mostly corresponding to rRNA operons, are left unresolved. The pBtic235 node is indicated in red. Dashed insets indicate enlargements of portions of assembly graphs close to the element-to-chromosome connection. (d) A simplified genetic map of pBtic235 is shown on the right. Circles from the centre represent: 1, scale (0 to 235.4 kb); 2, GC-skew; 3, G+C-content deviation from the mean; 4, CDS map; 5, coverage (black) with Illumina reads for the 4Q7_KBC_ sample; 6, coverage (blue) with Illumina reads for 4Q7_AS_; 7, coverage (green) with Illumina reads for 4Q7_JM_; 8, coverage (red) with ONT reads for 4Q7_JM_; 9, scale and selected genetic markers for the plasmid-like module [13] of the element. For convenience, linear vertical value scales are indicated for each read distribution. The same read distributions are presented in linear form on the left. Dashed lines indicate the correspondence of regions with overcoverage in the linear and circular presentations of the distributions. The regions are identical for 4Q7_AS_ and 4Q7_KBC_. Dashed arrows show the location of element-to-chromosome linkages confirmed with multiple sequencing reads. Note the gradual increase in coverage at around 150 and 100 kb, for the 4Q7_JM_ and 4Q7_AS_, and 4Q7_KBC_ samples, respectively, presumably due to the use of different active origins of replication. For 4Q7_KBC_, the distribution corresponds to the sequencing reads from SRR1174235 [1]. The distributions for 4Q7_AS_ and 4Q7_JM_ were generated from the reads obtained during this study (SRR11567778 and SRR11565157). NGS, Next Generation Sequencing.

We were unable to obtain an unambiguous automatic assembly of the region around the chromosomal replication termination site, even with the long reads provided by ONT technology. Therefore, we performed a semi-manual assembly graph analysis with Bandage software. Examples of this analysis, for all three samples of the *B. thuringiensis* serovar *israelensis* 4Q7 strain, are provided in Fig. 4. Bandage can be used for the selection of concrete assembly pathways from possibilities provided by an automatic assembler, taking into account such information as read coverage, detaching nodes (contigs) in situations in which multiple possible edges (links) are suggested, and by using homology information to guide solutions [26]. Thus, this software enables the expert to choose the most probable unique assembly pathway. Based on this analysis, we concluded that two of strain samples analysed, 4Q7_AS_ and 4Q7_KBC_, have extremely similar, if not identical, chromosomes (Fig. 4a, b). By contrast, 4Q7_JM_ has lost a small amount of DNA containing repeated sequences in the vicinity of the chromosomal terminus of replication (Fig. 4c). Moreover, the connection of the chromosomal part of the assemblies to the pBtic235 element also differed greatly between 4Q7_JM_ and the other two samples (Fig. 5a, b, c, d).

For all strain samples, we found that assembly of the chromosomal part of the genome suggested the existence of a linear form, presumably delimited by parts of the pBtic235 element at the extremities. We observed no multiple read-through ONT sequences that would provide a straightforward confirmation of chromosome circularization, providing indirect support for the possibility of chromosome linearity. However, additional direct reliable evidence is required before any firm conclusions can be drawn on this point.

## Discussion

Our analysis of the available sequencing reads for the genome of a model *B. thuringiensis* serovar *israelensis* 4Q7 strain revealed an unusual read distribution, indicating the occurrence of unexpected major genome-restructuring events during the construction or maintenance of this strain. We demonstrate here that, in addition to the loss of plasmids, prophages and a large (492 kb) region of the chromosome including the termination sequence for replication, the *B. thuringiensis* serovar *israelensis* 4Q7 strain has undergone several other chromosome-restructuring events. Our results indicate that, in the 553 kb area extending from the 2745 to 3298 kb positions in the reference genome *B. thuringiensis* serovar *israelensis* AM65-52, genome sequencing coverage for the 4Q7 strain gradually increases to twofold. We interpret this as indicating the occurrence of a duplication, resulting in a very long inverted repeat. In addition to the deletion of three putative prophages, each about 40 kb long, this duplication probably equalizes the sizes of the two chromosomal replichores, each extending from the replication origin to the new replication termination area.

The 492 kb deletion removes the region corresponding to the chromosomal replication termination site in the precursor strain, which has been formally defined as an area of GC-skew sign switching [32]. It is unclear how this loss is functionally compensated in the *B. thuringiensis* serovar *israelensis* 4Q7 strain. We suggest that replication is terminated at several different sites on the new chromosome, within the duplicated region. An intriguing hypothesis concerns the possible role of the extrachromosomal element pBtic235 in the termination of chromosomal replication, given that this element encodes its own replication termination protein and XerC recombinase, and the corresponding genes are located close to the border with the region displaying overcoverage during sequencing (corresponding to 225 kb; Fig. 3). The read distributions over the pBtic235 element are different between the three 4Q7 strain samples studied and the environmental strain ATCC 35646, which contains the standard, albeit incomplete, natural *B. thuringiensis* serovar *israelensis* plasmid set (environmental strain ATCC 35646 lacks the toxin-encoding plasmid, pBtoxis). We detected multiple reads joining the bacterial chromosome and the DNA of the pBtic235 element (Figs 2b and 5). However, it is impossible to determine, from our data, whether pBtic235, or part of this element, was simply integrated into the chromosome. Moreover, a recent publication reported that 4Q7 could be cured of this element, and pBtic235 was itself identified as a separate band on gel electrophoresis [13]. A natural *B. thuringiensis* serovar *israelensis* strain was also recently sequenced and found to lack a pBtic235 element [37]. Further studies of 4Q7 and strains similar to GSX002, the 4Q7 derivative cured of pBtic235 [13], should provide an explicit model for the complete genome structure and termination of chromosomal DNA replication in these strains.

Formally, our identification of reads connecting the chromosomal and pBtic235 element contigs can be illustrated by a Bandage analysis of the Bruijn graph corresponding to SPAdes *de novo* assembly (Fig. 4). The optimized automatic assembly of short Illumina reads provides a complex picture, with nodes that are clearly of double width, corresponding to the doubling of coverage. The node corresponding to pBtic235 is connected to this structure in the form of a loop, suggesting that there are reads joining them. The manual optimization of assembly, with Bandage, and the splitting of this node with double coverage into two and the detachment of pBtic235, result in a fairly good assembly of the chromosome, albeit different in the 4Q7_JM_ strain variant and the others, 4Q7_AS_ and 4Q7_KBC_ (Fig. 4c).

We currently consider the best assembly of the data for the *B. thuringiensis* serovar *israelensis* 4Q7_JM_ sample obtained, at least in terms of reproducibility, to be that obtained by the automatic application of Unicycler software to different partial sets of the ONT data and all the Illumina data. This conclusion is supported by the finding that the 4Q7_AS_ and 4Q7_KBC_ samples are clearly different from the 4Q7_JM_ sample. We interpret these differences between samples to be due to rapid evolution of the genome of this strain. The assembly for the 4Q7_JM_ sample lacks the 8015 bp contig and several smaller contigs, but there are also several other small differences between the sequenced samples. The proposed version of the genome assembly is sufficient for many practical purposes involving the experimental use of this strain. However, studies of the rapid evolution of genome structure, including other experimental methods, such as PFGE, would be required to infer the correct, stable sequence.

The issues of chromosomal replication termination in *B. thuringiensis* serovar *israelensis* strains and the relationship with the enigmatic pBtic235 element remain intriguing. This non-integrated prophage [13] is the only extrachromosomal element persisting in the 4Q7 strain with no obviously profitable function for the host, after multiple treatments [12, 14]. This element can be removed by targeted elimination [13]. Interestingly, a component of the chromosomal replication termination model, an analogue of the *Bacillus subtilis* protein Rtp, which is thought to be the functional counterpart of the *E. coli* protein Tus in Gram-positive bacteria [38, 39], does not seem to exist in the *B. cereus* group. However, pBtic235 encodes a protein, BTF1_31667, displaying some similarity (48 % identity) to Rtp [13]. It could be speculated that pBtic235 provides the chromosome with a means of terminating replication, as we detected an association of the chromosome with pBtic235. Slight over-replication was observed, in the form of a higher than average coverage of the genome in terms of the number of sequencing reads in the vicinity of this gene (Fig. 5d). If this protein plays an important role, then the chromosomal Rtp function should also be replaced by another protein in the *B. cereus* group. Likely candidates for this protein would include RecQ-family proteins, such as that encoded by the AND24642 locus in *B. thuringiensis* serovar *israelensis* AM65-52. A similar gene is also present at a similar chromosomal site, close to the site of replication termination, in *B. subtilis* (accession no. NP_389803), but this gene has not yet been implicated in the termination of replication.

Only 12 of the 26 minor variations between studied 4Q7 samples and AM65-52 genomes detected appeared to have arisen during the curing of plasmids, as they differed from other sequenced *B. thuringiensis* serovar *israelensis* strains and were common to all 4Q7 samples. Seven other variants were due to strain sample maintenance or other manipulations, as they were unique to one of the three samples used. The 4Q7_KBC_ sample contained only variants common to the other two 4Q7 samples. It should, therefore, correspond to the strain sample in the BGSC collection. The list of mutated genes and the nature of the amino-acid changes encoded did not indicate that any of these mutations would be likely to provide 4Q7 with new properties. Therefore, we assume that only the genome-restructuring events were likely to have played an important role in adaptation to plasmid-curing conditions.

The natural occurrence of long inverted repeats close to the replication termination site in bacteria with circular chromosomes appears to be rare, based on a recent analysis of 1373 sequenced genomes [40]. According to the authors, among bacteria that had not been manipulated artificially, this property was found only in the branch of *Lactobacillus delbrueckii* designated subsp*. bulgaricus*.

In the case described here, the *B. thuringiensis* serovar *israelensis* 4Q7 strain was not selected for this property, although the treatment of its precursors would certainly have impaired plasmid replication, and the curing of naturally acquired plasmids was tested in these precursors. The most intriguing features of this strain are the large size of the repeats (553 kb, versus only 38 kb in *L. delbrueckii* subsp* . bulgaricus* [40]) for each copy of the duplicated region, and the loss of its natural replication termination machinery, for which the *dif* and *xerCD* genes were probably the most important elements [32]. The aforementioned analysis of 1373 genomes [40] dealt only with deposited assemblies and not with raw sequencing data. We investigated the *B. thuringiensis* and *B. cereus* genomes further, by applying a read alignment protocol to a set of 91 raw sequencing reads for *B. thuringiensis* and 480 for *B. cereus *, using the *B. thuringiensis* serovar *israelensis* AM65-52 chromosome as the alignment template. We were able to detect only one set of reads, DRR002381, in BioSample SAMD00015926, corresponding to the *B. thuringiensis* LDC-391 strain, which is cytotoxic to human cancer cells [2, 3], indicating that this strain may also carry a large genomic duplication in the vicinity of its replication termination site (Fig. S5). This analysis is very preliminary but, nevertheless, it suggests that the duplication event reported here is rare, but not unique, in the genomes of the *B. cereus* group.

The full impact of structural changes to the genome on bacterial physiology and virulence in *B. thuringiensis* serovar *israelensis* 4Q7 remains unclear, but several potential consequences can be foreseen. First, the deletion of the prophage region 2 (3307 to 3451 kb in AM65-52), previously characterized under the name phIS3501 [14, 41], leads to the formation of intact gene for the haemolytic toxin HlyII. The stable deletion of this prophage, therefore, leads to the formation of a potentially important virulence factor gene. In addition, the 553 kb duplication described here encompasses the gene encoding the Nhe toxin, another known virulence factor [42]. Hence, the duplication would lead to a proportional increase in gene expression and, thus, in virulence related to this factor. In addition, *B. thuringiensis* serovar *israelensis* 4Q7 and the natural strains of this lineage appear to have a very different chromosomal gene content, as shown by the previously detected 492 kb deletion [1]. Conclusions about the behaviour of this strain when used as a model for physiological or virulence studies, therefore, should be drawn and interpreted with caution.

## Supplementary Data

Supplementary material 1
