# Supplementary material for: Long inverted repeats around the chromosome replication terminus in the model strain Bacillus thuringiensis serovar israelensis BGSC 4Q7
Source: Microb Genom. 2020 Nov 12;6(12):mgen000468. doi: 10.1099/mgen.0.000468 (PMC8116677; doi:10.1099/mgen.0.000468)
Supplement: Supplementary material 1 [file mgen-6-468-s001.pdf]

# **Long inverted repeats around replication terminus of the chromosome in model strain *Bacillus thuringiensis* serovar *israelensis* BGSC 4Q7.**

Alexander Bolotin[1], Benoit Quinquis[2], Hugo Roume[2], Michel Gohar[1], Didier Lereclus[1] and Alexei Sorokin[1]*

[1]Université Paris-Saclay, INRAE, AgroParisTech, Micalis Institute, 78350, Jouy-en-Josas, France

[2]MetaGenoPolis, INRAE, 78350, Jouy-en-Josas, France

# Supplementary Figure legends

**Figure S1. Correlation of *Illumina* reads' coverage with GC content.**

The phenomenon studied earlier (Dohm et al., 2008; Minoche et al., 2011) of correlation of NGS reads' coverage with GC content of genomes is illustrated. The distribution images over pBtic235 element sequence were copied from the Tablet interface panel and slightly modified for more visible representation. (A) reads for *Bti* $4Q7_{KBC}$ from SRR1174235 (Jeong et al., 2014) and other indicated samples of *Bti* strains, obtained from *Eurofins* GATC in 2019. (B) reads for *Bti* AM65-52 and *Bti* ATCC35646 generated by *Eurofins* GATC in 2017. (C) GC-content distribution for pBtic235, window is 2 Kb, step is 0.5 Kb. The vertical scales are linear, the values are not shown. The horizontal scale in kb is drawn below. Note visible correlation of GC-content distribution with read coverage for the strains *Bti* AM65-52 and *Bti* ATCC35646. Apparently the correlation is due to library construction protocol and/or the used algorithm of base-calling software.

**Figure S2. *Illumina* reads aligned over *Bti* AM65-52.**

Distribution of *Illumina* sequencing reads, in coverage per nucleotide, for *Bti* $4Q7_{KBC}$ (top), *Bti* $4Q7_{AS}$ (middle) and *Bti* $4Q7_{JM}$ (bottom) samples of *Bti* 4Q7 strain over the *Bti* AM65-52 genome (acc. # CP013275). Only 800 Kb part of the whole distribution is shown, spreading from 2,640 to 3,440 Kb area. Non-covered regions correspond to parts of the 492 Kb area and prophage Region 2, deleted in *Bti* 4Q7. The duplicated 553 Kb area from 2,745 to 3,298 Kb corresponds to elevated read coverage. The distribution image was copied from the Tablet interface panel and slightly modified for more visible representation. The vertical and horizontal

scales are linear, the values are not shown. The *Bti* 4Q7$_{KBC}$ distribution corresponds to alignment done using sequencing reads SRR1174235 (Jeong et al., 2014). The reads for samples *Bti* 4Q7$_{AS}$ and *Bti* 4Q7$_{JM}$ were generated during the current study (SRR11567778 and SRR11565157).

**Figure S3. Size distribution of *MinION* reads produced for the *Bti* 4Q7$_{JM}$ sample.**

Semi-logarithmic scale is used for better graphical presentation. Conversion Table is shown on the right and scale in kilobases - under the logarithmic diagram scale.

**Figure S4. *MinION* reads vs *Illumina* reads in an assembly.**

Alignment of *MinION* (top) and *Illumina* (bottom) reads over a part of *Bti* AM65-52 genome sequence as template is shown as presented by the Tablet (Milne et al., 2013) assembly browser. Grey and rose spots indicate that the read character corresponds or not to that of template, respectively.

**Figure S5. Duplicated region in the genome of *B. thuringiensis* LDC-391 strain.**

Distribution of *Illumina* sequencing reads over the *Bti* AM65-52 genome (acc. # CP013275), in coverage per nucleotide, are shown for *Bt* LDC-391 (top), *Bti* 4Q7$_{KBC}$ (middle) and *Bti* HD1002 (bottom) corresponding to raw data DRR002381, SRR1274235 and SRR2175483, respectively. Only 2.1 Mb part of the whole distribution is shown, spreading from 1,950 to 4,050 Kb area. The *Bti* distributions are shown only for comparison with the *Bt* LDC-391. The over-covered region

from 2.6 to 3.4 Mb in the *Bt* LDC-391 distribution may correspond to an 800 Kb duplication. Non-covered regions near 3,550 and 3,800 Kb correspond to prophage Regions 2 and 5 detected earlier (Gillis et al., 2018), and thus deleted in the *Bt* LDC-391 strain. It is notable also elevated coverage of the prophage Region 5 in *Bti* HD1002.

# References

Dohm, J.C., Lottaz, C., Borodina, T., and Himmelbauer, H. (2008). Substantial biases in ultra-short read data sets from high-throughput DNA sequencing. Nucleic Acids Res *36*, e105.

Gillis, A., Fayad, N., Makart, L., Bolotin, A., Sorokin, A., Kallassy, M., and Mahillon, J. (2018). Role of plasmid plasticity and mobile genetic elements in the entomopathogen Bacillus thuringiensis serovar israelensis. FEMS Microbiol Rev *42*, 829-856.

Jeong, H., Park, S.-H., and Chol, S.-K. (2014). Genome Sequence of the Acrystalliferous Bacillus thuringiensis Serovar Israelensis Strain 4Q7, Widely Used as a Recombination Host. Genome Announcements *2*, e00231-00214.

Milne, I., Stephen, G., Bayer, M., Cock, P.J., Pritchard, L., Cardle, L., Shaw, P.D., and Marshall, D. (2013). Using Tablet for visual exploration of second-generation sequencing data. Briefings in bioinformatics *14*, 193-202.

Minoche, A.E., Dohm, J.C., and Himmelbauer, H. (2011). Evaluation of genomic high-throughput sequencing data generated on Illumina HiSeq and genome analyzer systems. Genome Biol *12*, R112.

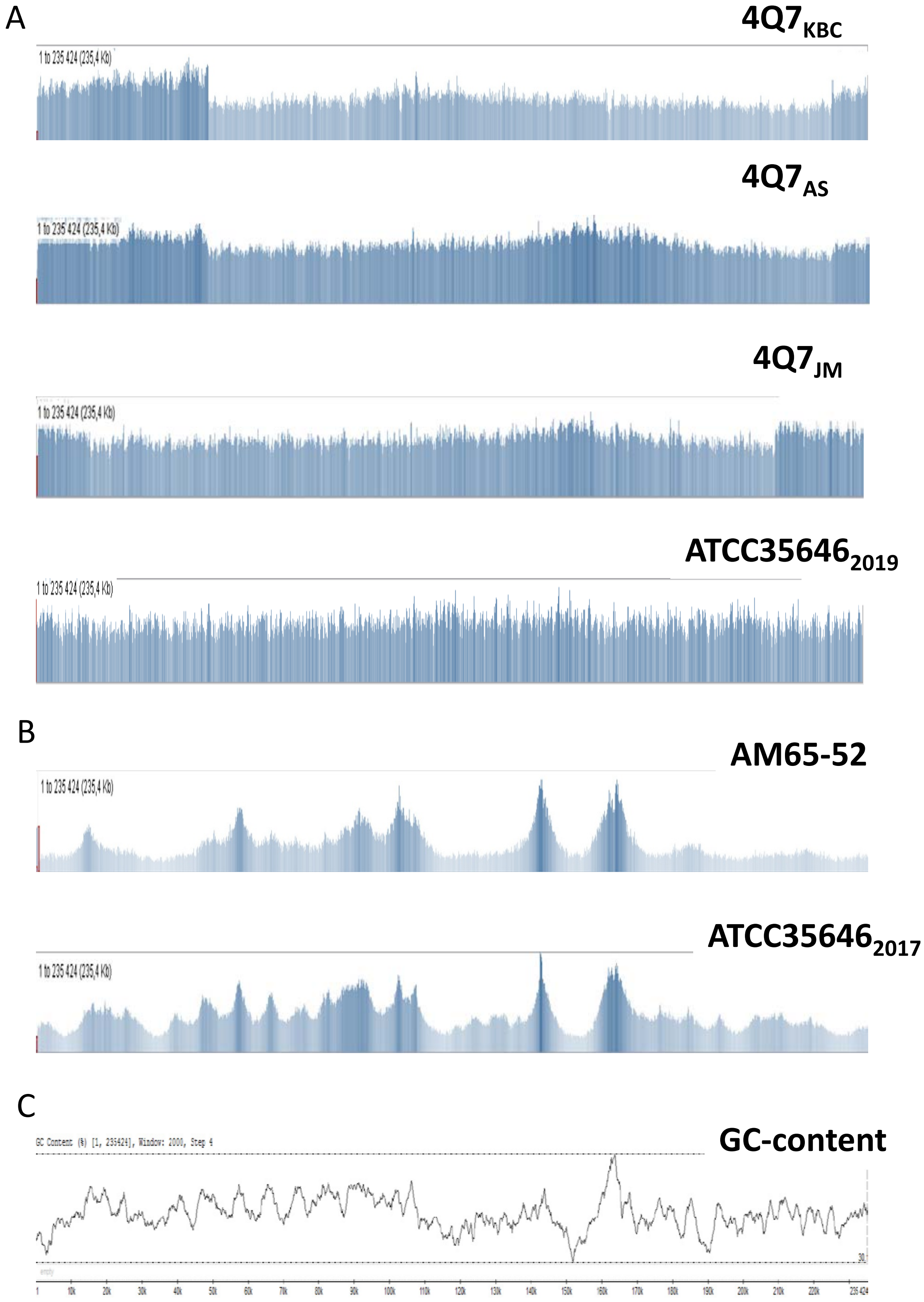
A
4Q7KBC
1 to 235 424 (235,4 Kb)
4Q7AS
1 to 235 424 (235,4 Kb)
4Q7JM
1 to 235 424 (235,4 Kb)
ATCC35646 2019
1 to 235 424 (235,4 Kb)
B
AM65-52
1 to 235 424 (235,4 Kb)
ATCC35646 2017
1 to 235 424 (235,4 Kb)
C
GC-content
GC Content (%) [1, 235424], Window: 2000, Step 4

Figure S1

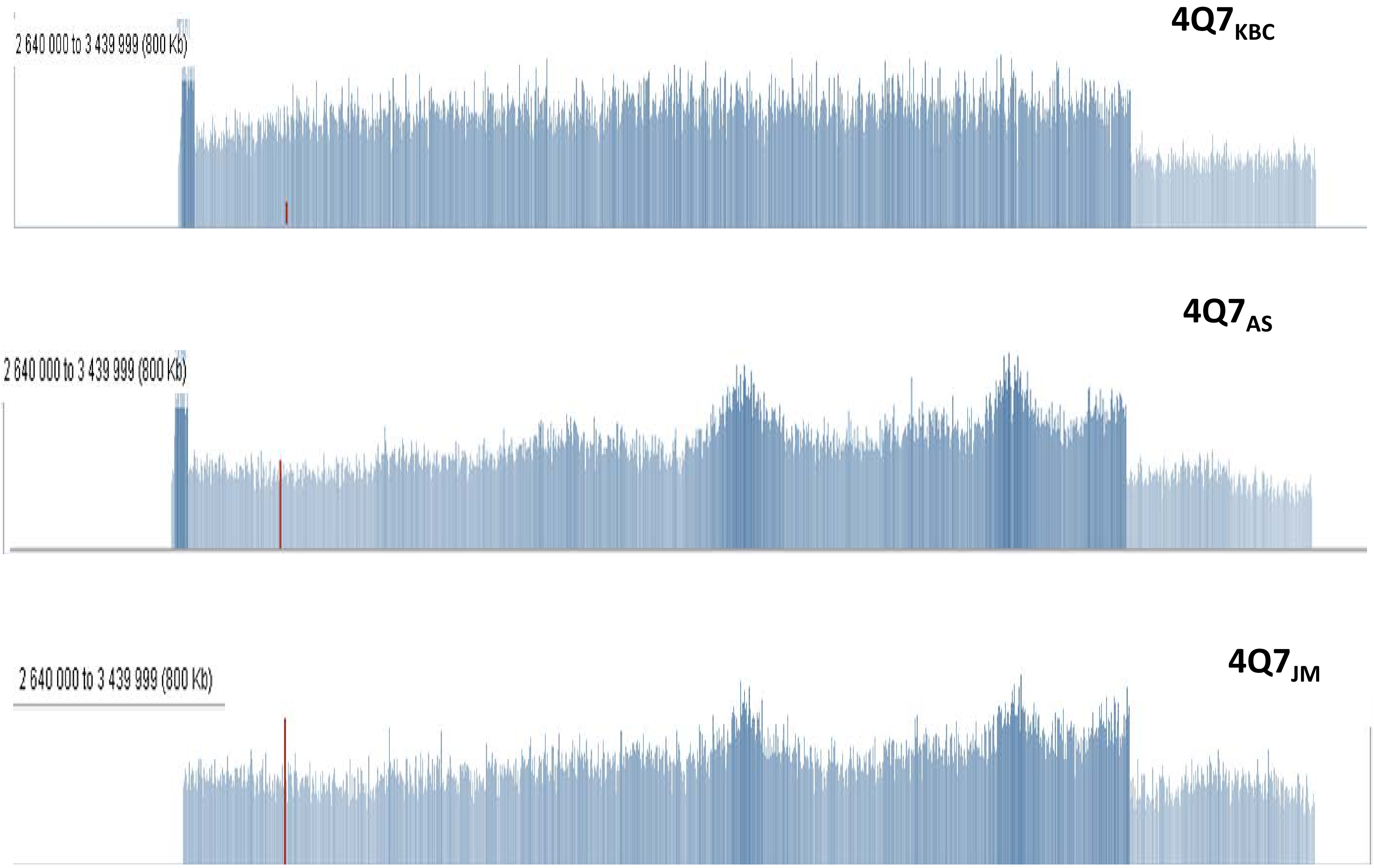


Figure S2. *Illumina* reads for different samples of *Bti* 4Q7aligned over *Bti* AM65-52 genome

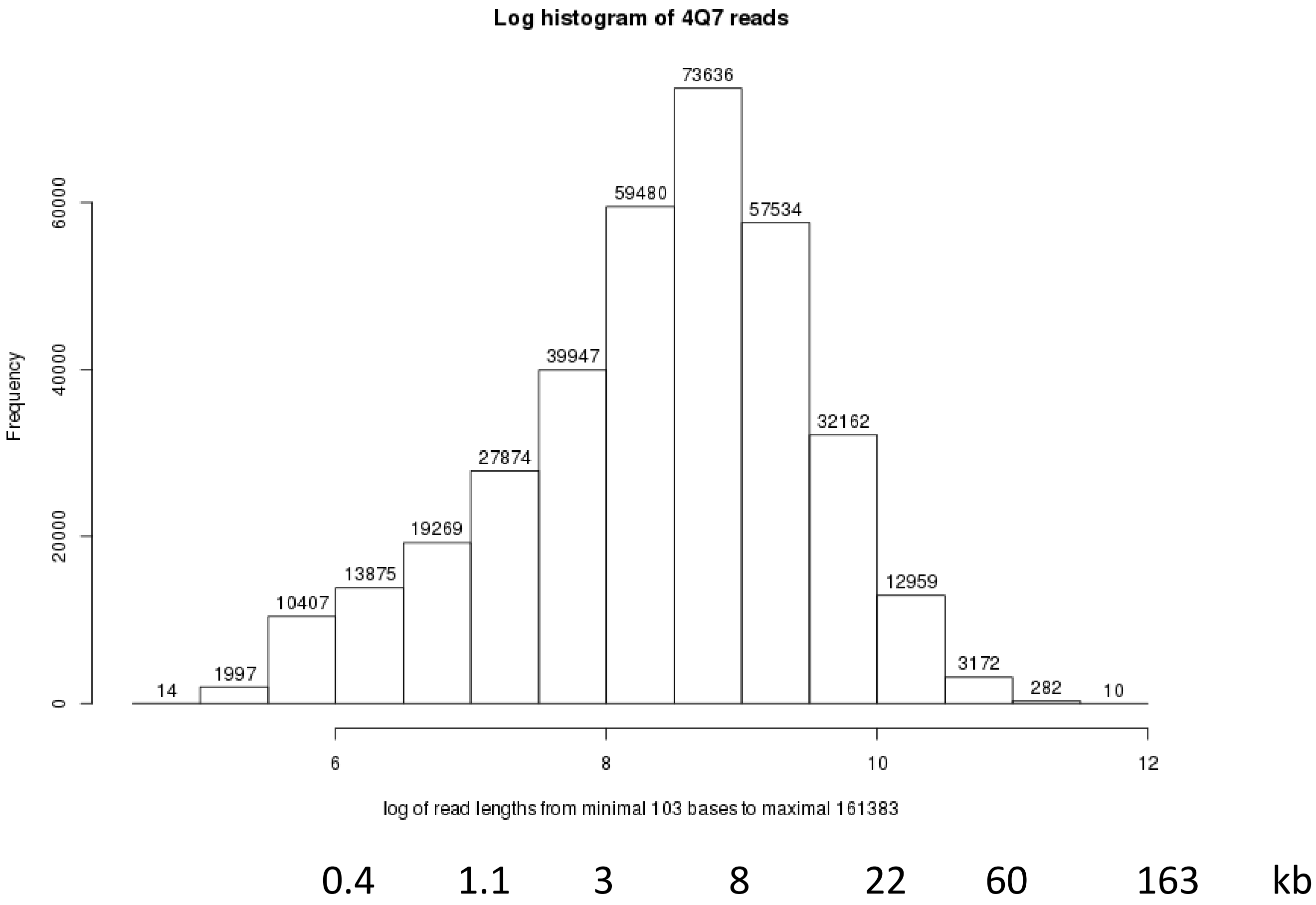


| log | bases |
|---|---|
| 5 | 148 |
| 5.5 | 245 |
| 6 | 403 |
| 6.5 | 665 |
| 7 | 1,097 |
| 7.5 | 1,808 |
| 8 | 2,981 |
| 8.5 | 4,915 |
| 9 | 8,103 |
| 9.5 | 13,360 |
| 10 | 22,026 |
| 10.5 | 36,316 |
| 11 | 59,874 |
| 11.5 | 98,716 |
| 12 | 162,755 |

Figure S3. Size distribution of *MinION* reads for the 4Q7$_{JM}$ sample

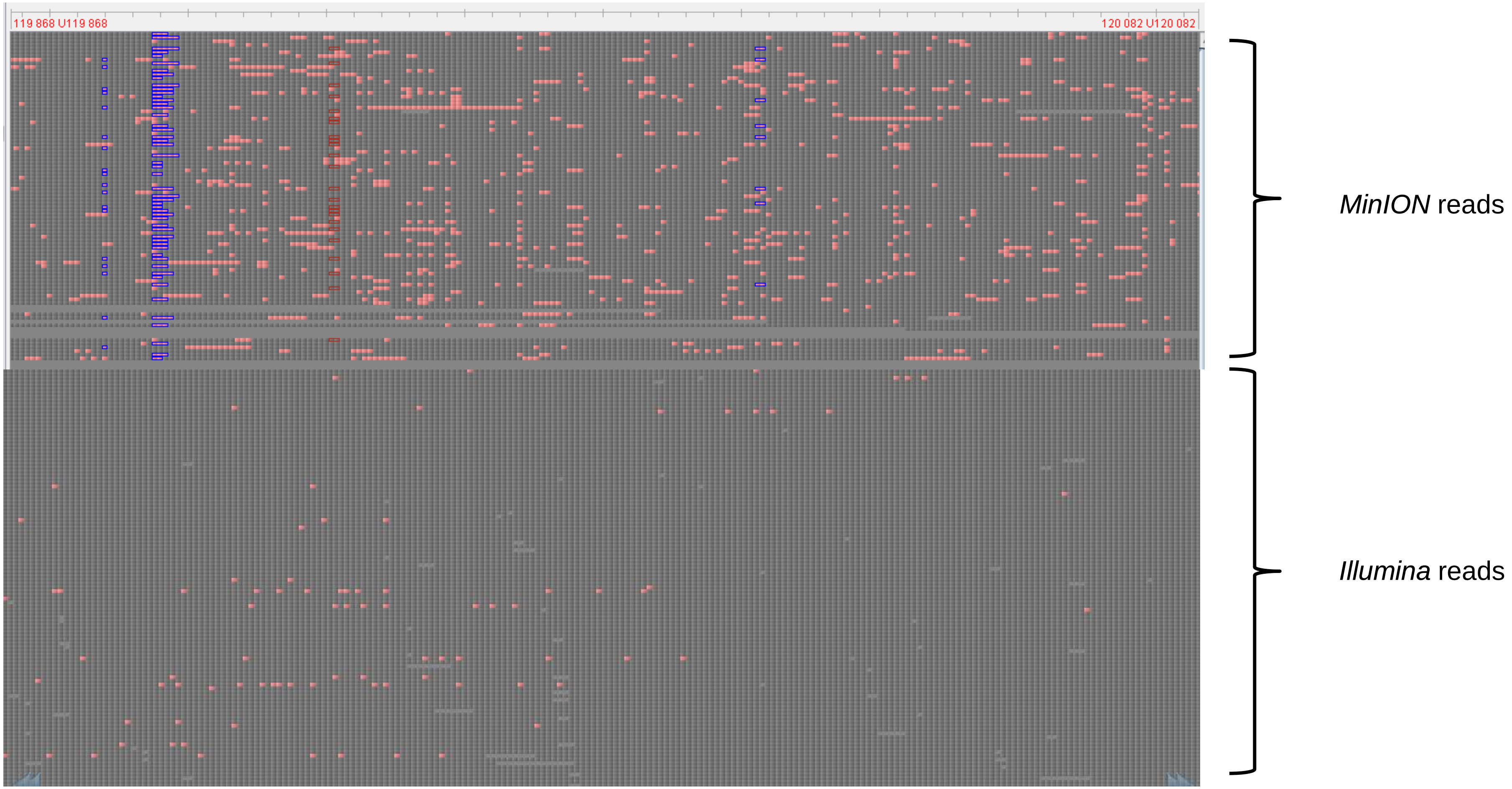


Figure S4. *MinION* reads vs *Illumina* reads in an assembly

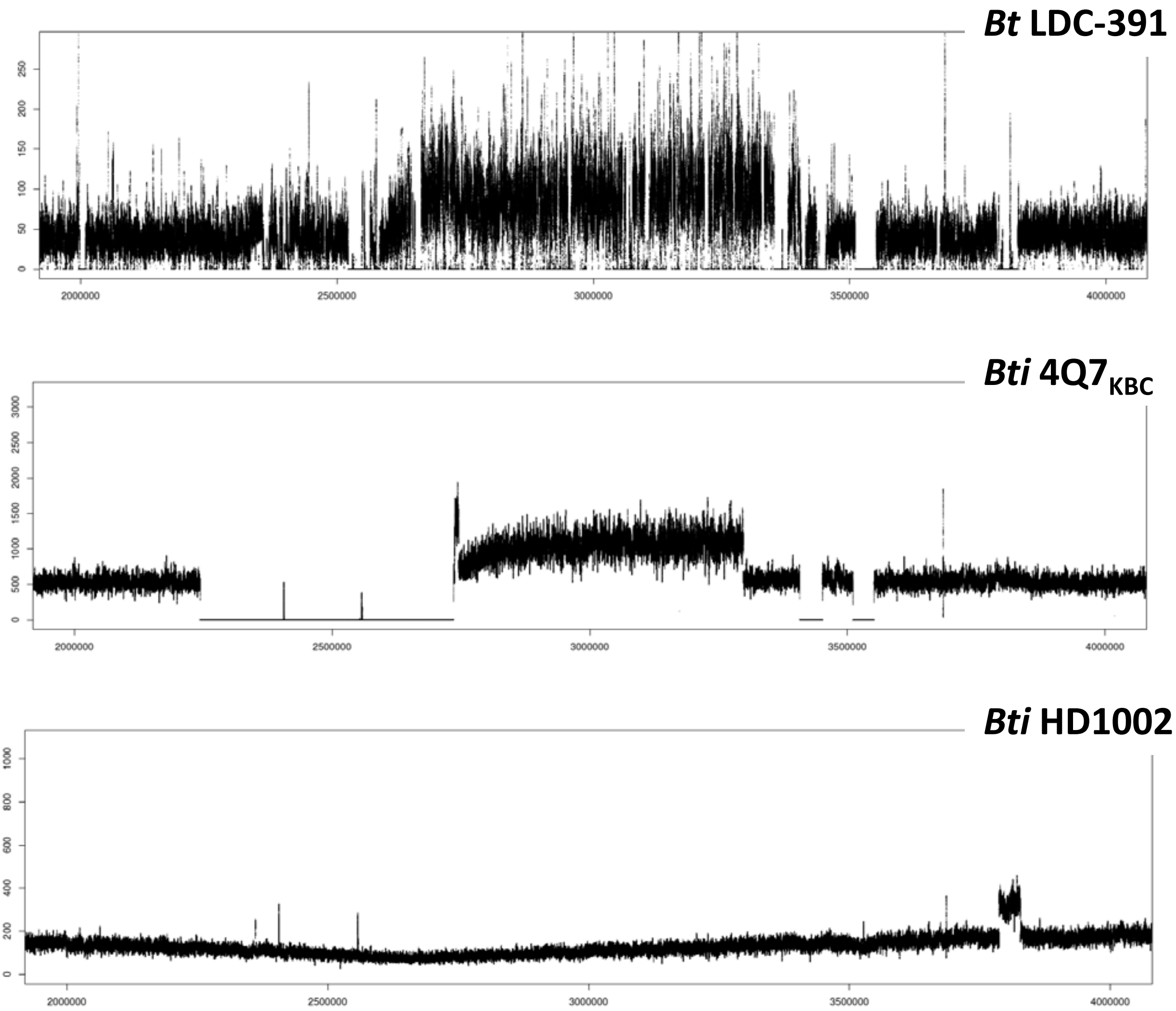


Figure S5. Duplicated region in the genome *of B. thuringiensis* LDC-391 strain.
